# Surgical management of chronic lateral ankle instability: a meta-analysis

**DOI:** 10.1186/s13018-018-0870-6

**Published:** 2018-06-25

**Authors:** Yongxing Cao, Yuan Hong, Yang Xu, Yuan Zhu, Xiangyang Xu

**Affiliations:** 1 Department of Orthopedics, Ruijin Hospital North, Shanghai Jiao Tong University School of Medicine, Shanghai, China; 2 Department of Orthopedics, Ruijin Hospital, Shanghai Jiao Tong University School of Medicine, Shanghai, China

**Keywords:** Ankle sprain, Ankle instability, Lateral ligament injury, Surgical treatment, Meta-analysis

## Abstract

**Background:**

A key point to surgical treatment of chronic lateral ankle instability is choosing a suitable surgical procedure. The purpose of this meta-analysis was to compare different surgical techniques for management of chronic lateral ankle instability.

**Methods:**

We searched the Cochrane Library, MEDLINE, and EMBASE. All identified randomized and quasi-randomized controlled trials of operative treatment for chronic lateral ankle instability were included. Two review authors independently extracted data from each study and assessed risk of bias. Where appropriate, results of comparable studies were pooled.

**Results:**

Seven randomized controlled trials were included for analysis. They fell in five clearly distinct groups.

One study comparing two different kinds of non-anatomic reconstruction procedures (dynamic and static tenodesis) found two clinical outcomes favoring static tenodesis: better clinical satisfaction and fewer subsequent sprains.

Two studies compared non-anatomic reconstruction versus anatomic repairment. In one study, nerve damage was more frequent in non-anatomic reconstruction group; the other one reported that radiological measurement of ankle laxity showed that non-anatomic reconstruction provided higher reduction of talar tilt angle.

Two studies comparing two anatomic repairment surgical techniques (transosseous suture versus imbrication) showed no significant difference in any clinical outcome at the follow-up except operation time.

One study compared two different anatomic repairment techniques. They found that the double anchor technique was superior with respect to the reduction of talar tilt than single anchor technique.

One study compared an anatomic reconstruction procedure with a modified Brostrom technique. Primary reconstruction combined with ligament advanced reinforcement system results in better patient-scored clinical outcome, at 2 years post-surgery, than the modified Brostrom procedure.

**Conclusions:**

There is limited evidence to support any one surgical technique over another surgical technique for chronic lateral ankle instability, but based on the evidence, we could still get some conclusions: (1) There are limitations to the use of dynamic tenodesis, which obtained poor clinical satisfaction and more subsequent sprains. (2) Non-anatomic reconstruction abnormally increased inversion stiffness at the subtalar level as compare with anatomic repairment. (3) Multiple types of modified Brostrom procedures could acquire good clinical results. (4) Anatomic reconstruction is a better procedure for some specific patients.

**Electronic supplementary material:**

The online version of this article (10.1186/s13018-018-0870-6) contains supplementary material, which is available to authorized users.

## Background

Acute lateral ankle ligament injury is one of the most common problems in foot and ankle medicine [1]. The lateral ankle ligament complex consists of the anterior talofibular ligament (ATFL), the calcaneofibular ligament (CFL), and the posterior talofibular ligament (PTFL). The weakest of the three lateral ankle ligaments is the ATFL, which is the most frequently injured in ankle sprains, whereas the CFL is involved in 50~75% of such injuries, and the PTFL in < 10% [2]. The ATFL rupture is evaluated by the anterior drawer test, which is generally classified as mild, moderate, and severe degree. The CFL is rarely injured alone, but is associated with ATFL tears in more severe injuries. The CFL rupture is evaluated by the talar tilt test and is corroborated with a stressed anteroposterior radiograph. The PTFL is the strongest ligament of the lateral ligament complex and is rarely injured [3]. After injury, initial treatment is usually conservative, such as functional rehabilitation. An incidence of 10–30% of patients will fail conservative treatment, result in chronic ankle instability (CAI), and require surgical management [4].

To date, many surgical procedures have been described to manage chronic ankle instability, indicating the complexity of the current status. These procedures and their modifications fall into the following three categories: non-anatomic reconstruction, anatomic repairment, and anatomic reconstruction [1].

Non-anatomic reconstruction procedures use various configurations of local tendon grafts to accomplish the restriction function of the ligament without repair of the ligament remnants. Several techniques have been described, including partial or complete tenodesis from the Achilles tendon or peroneal tendon [5] or allografts mimicking the function of the lateral ankle ligaments such as the Chrisman-Snook (CS) procedure, the Watson-Jones procedure, and the modified Evans procedure [6–8].

Anatomic repairment is to restore normal anatomy and joint mechanics by in situ repair of the injured ligament. Anatomic repairment includes repair ligaments by either shortening and fixing them to the bone surfaces or augmenting them with local structures to enhance the repairment. A typical example is the Brostrom-Gould procedure [9], which enhances the original ligaments with the extensor retinaculum and has proved to be a strong procedure without sacrificing other normal structures [5].

Anatomic reconstruction procedures use tendon grafts to recreate joint biomechanics anatomically by replicating the anatomic positions of the ATFL and CFL origin and insertion sites. They vary in the means by which they attain that positioning, including the number and angle of tunnels in the fibula and the fixation techniques selected in each bone tunnel location [1, 10–13].

A key point to surgical treatment of chronic lateral ankle instability is choosing a suitable surgical procedure, which is a complex question that has many arguments in the theory and clinical practice. The biggest limiting factor is that there are few high-quality controlled trials available to assist foot and ankle surgeons in making an informed decision.

The purpose of this meta-analysis was to perform an extensive review of the literature systematically and to attempt to compare different surgical techniques for management of chronic lateral ankle instability.

## Methods

This meta-analysis was reported according to the preferred reporting items for systematic reviews and meta-analysis guidelines. All analyses were based on previous published studies; thus, no ethical approval and patient consent are required.

### Criteria for considering studies for this review

#### Eligible trial design

Any randomized and quasi-randomized (methods of allocating participants to a treatment which is not strictly random, e.g., by date of birth, hospital record number, alternation) controlled clinical trial evaluating any surgical treatments for chronic lateral ankle instability in adults was considered for inclusion. Chronic lateral ankle instability was defined as symptoms of lateral ankle instability, giving way or recurrent sprains, persisting for more than 6 months.

#### Patient characteristics

Studies including adult participants with chronic lateral ankle instability who underwent a surgical intervention were included. Trials containing adults and children were included if separate data for adults could be obtained. Studies evaluating exclusively with people with congenital deformities or children or degenerative conditions were excluded. Studies dealing exclusively with the prevention of ankle sprains in healthy individuals or conservative intervention of acute injury to the lateral ankle ligaments were also excluded.

#### Intervention-comparator characteristics

Trials comparing different types of surgical treatment used for treating chronic lateral ankle instability were included. We planned to include all three major types of surgical interventions (non-anatomic reconstruction, anatomic repairment, and anatomic reconstruction) and compare the clinical effect of different operative approaches. In this article, we define anatomic repairment as the primary or secondary suturing of the torn lateral ligaments at their anatomic position. The Brostrom procedure is a true repairment of the lateral ligaments. The classic Brostrom procedure is rarely performed as an operative technique alone. It is usually augmented with the mobilized lateral portion of the extensor retinaculum or periosteal flap. This kind of procedure is considered as a modified Brostrom procedure (MB). The procedure may be performed in the traditional manner through drill holes or with bone anchors [9, 14–16]. We define reconstruction as the replacement of the chronically deficient lateral ligaments with local tissues or with autograft or allograft tissue. Anatomic reconstruction is placement of the transferred tendon grafts in such a way as to replicate the anatomic positions of the ATFL and CFL origin sites. These procedures vary in the means of the fixation techniques selected in each bone tunnel location. The position, number, and angle of tunnels are varied, too. Non-anatomic reconstruction stabilizes the ankle using tendon grafts placed non-anatomically, such as the CS, Evans, and Watson-Jones procedure. In reconstruction procedures, there are many different ways the ligament graft can be secured in the bone including anchors, bone tunnels with interference screws, and endobutton-type devices [10–13].

#### Clinical state

##### Subjective evaluation of symptoms

1. Satisfaction after operation: For the assessment of clinical outcomes post-operation, the Karlsson score and/or FAOS (foot and ankle outcome score) and/or the Sefton grading system were used. The Karlsson score and FAOS are methods of evaluating improvement in postoperative function and outcome by examining the stability of the ankle joint, pain, swelling, range of motion, activities at work or during sports, activities of daily living, the ability to climb stairs, running ability, and the use of ankle support aids. The Sefton grade was measured postoperatively at the follow-up visit and was classified as excellent, good, fair, or poor. Grades greater than good were considered as satisfactory treatment results.
2. Subjective instability, pain, and swelling: Some studies did not use the grading system to assess the functional outcomes, but instead provide the number of patients with subjective instability, pain, swelling, and so on.

##### Clinical measurements

For clinical assessment, the anterior drawer and varus stress radiography were measured preoperatively and at the follow-up visit in most studies. We collected preoperative and postoperative data of anterior talar translation and the talar tilt angle for comparison.

##### Complications

We collected data for intraoperative complications (drill hole fracture, breakage of the anchor, etc.) and postoperative ones (wound complications, nerve damage, stiffness, subsequent sprains, deep vein thrombosis, revision, etc.)

### Search methods for identification of studies

We searched the Cochrane Library (to December 2016), MEDLINE (1990 to December 2016), and EMBASE (1990 to December 2016). We did not apply any language restrictions. In MEDLINE (PubMed online), a subject-specific strategy was combined with the Cochrane Highly Sensitive Search Strategy for identifying randomized trials. Search strategies for Cochrane, MEDLINE, and EMBASE can be found in Additional file 1. We looked for reference lists of articles deemed eligible in the field to identify further studies or additional data. We also attempted to locate unpublished material or contact researchers for unpublished studies.

### Data collection

The titles and abstracts of all downloaded documents from the electronic searches were screened by CYX, who discarded clearly irrelevant reports. The remaining citations were then screened independently by CYX and HY to establish the need for obtaining full-text articles. Full-text articles were also obtained where there was any uncertainty about the relevance of the study. Subsequently, CYX and HY independently selected studies according to the inclusion criteria of the review. Disagreements were resolved by consultation and discussion with another review author (ZY).

Two review authors (CYX and HY) independently extracted data from each trial using a data extraction form and entered data into Review Manager 5.3 [17]. We recorded qualitative details and data regarding the study groups, interventions, and outcomes. Where necessary, we contacted trial authors for further details. Any differences in the data extraction between the review authors were resolved by discussion with a third review author (ZY).

### Evaluation of trial quality

Two review authors (CYX and HY) independently assessed the risk of bias in the included studies. Any differences were resolved by a consensus procedure, followed, if required, by scrutiny from a third review author (ZY). We used The Cochrane Collaboration’s “Risk of bias tool” [18]. Each study was graded for risk of bias in each of the following domains: adequate sequence generation, allocation concealment, blinding, incomplete outcome data addressed, free of selective outcome reporting, and free of other bias. Due to the nature of the interventions, assessors blinding was evaluated too.

### Data analysis

Treatment effect was measured using risk ratios and 95% confidence intervals for dichotomous data. Mean differences (MD), standardized mean differences (SMD), and 95% confidence intervals were calculated for continuous outcomes. When the measurement method or unit of the same intervention effect is exactly the same, MD was chosen. When different measurement methods or units are used for the same intervention effect, SMD was chosen.

Trail investigators were contacted for missing data if necessary. No studies with missing information on the variance measure were found. Where appropriate, we performed intention-to-treat analyses and were alert to the possibility of unreported loss to follow-up.

We combined trial results only where the participant groups, interventions, and outcome measures were sufficiently similar, as judged by clinical criteria and consideration of the statistical heterogeneity. Between-study variance was estimated using the DerSimonian and Laired estimator.

The data available from the included trials were insufficient to carry out our preplanned subgroup analyses (isolated ATFL repair versus ATFL and CFL repair; open versus mini-invasive approach) as well as a set of sensitivity analyses to examine the impact of the inclusion of trials with a high risk of bias, and also the effects of missing data on trial results.

## Results

### Description of studies

**Fig. 1:**
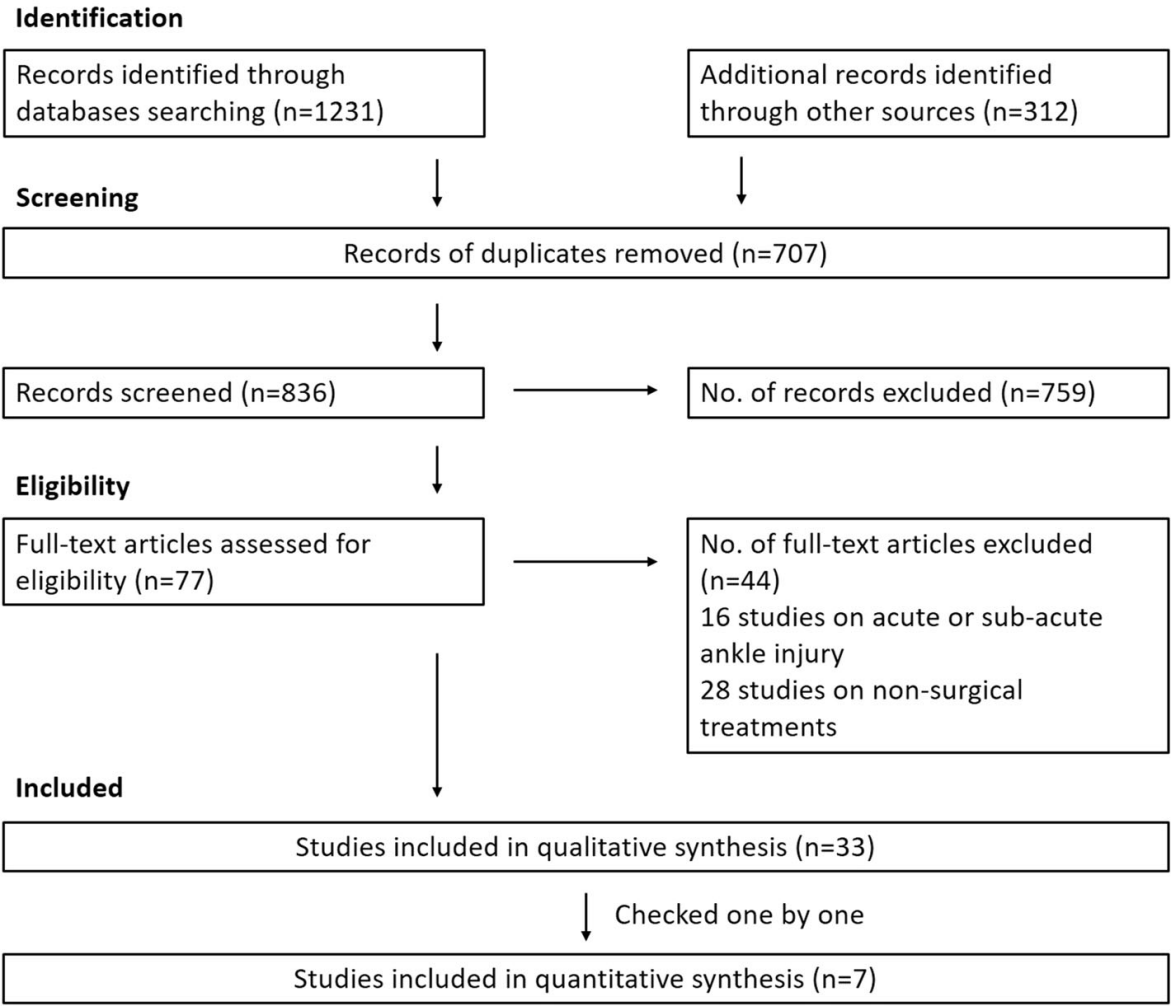
PRISMA 2009 flow diagram

We identified and screened a total 1231 records from the following databases: Cochrane Library (327), MEDLINE (593), and EMBASE (311). Another 312 articles were identified through screening the reference list of included studies. After removing duplicates, 836 titles and abstracts were reviewed. We identified no relevant trials from searching conference proceedings or the reference lists of the included studies. A total of 77 potentially trials were identified. We excluded a total of 16 studies on acute or sub-acute ankle injury [19–34] and 28 studies on non-surgical treatments [35–62]. The remaining 33 studies were eligible for further analysis (Fig. 1).

Seven studies were included for analysis [63–69]. All studies were published in English between 1990 and 2016 and identified in the Cochrane Library, MEDLINE, or EMBASE. All studies evaluated patients with CAI. They are summarized below, with a full summary for each trial detailed in the characteristics of included studies (Additional files 2, 3, 4, 5, 6, 7, and 8).

As expected, the unit of randomization was the individual patient in the included studies. There were no trials with a cluster-randomized design. None of the seven studies evaluated exactly the same criteria. Larsen [67] compared a dynamic tenodesis with a static tenodesis. Both procedures were non-anatomic reconstruction. The main outcome measures were a self-designed evaluation scheme and postoperative complications. Two studies compared an anatomic repairment with non-anatomic reconstruction [65, 69]. Hennrikus et al. [65] compared the outcome after a MB procedure with the CS procedure in 42 ankles. The main outcome measure was the Sefton ankle score, radiographic stability, and postoperative complications. Rosenbaum et al. [69] compared the outcome after a MB procedure with the Evans procedure in 20 participants. The main outcome measures were manual evaluation of joint mobility and radiographic stability. The other two studies compared a transosseous anatomic repairment procedure with an imbrication procedure [63, 66]. Cho et al. [63] compared a MB procedure with a transosseous suture procedure. The main outcome measure was Karlsson score, radiographic stability, and complications. Karlsson et al. [66] compared two different anatomic repairment in 60 patients. The lateral ankle ligaments were shortened by transosseous suture in one group. In the other group, the lateral ligaments were imbricated and reinforced by the inferior extensor retinaculum, a MB procedure. The main outcome measure was the Karlsson score, radiographic stability, and postoperative complications. Cho et al. [64] compared single anchor MB procedure with double anchor MB procedure in 50 patients. The main outcome measures were Karlsson score, radiographic stability, and complications. Porter et al. [68] compared a MB procedure with an anatomic reconstruction procedure with ligament advanced reinforcement system (LARS). The main outcome measures of Porter et al. [68] were FAOS and complications.

We excluded a total of 26 studies. Detailed reasons for exclusion can be found in the characteristics of excluded studies (Additional file 9).

### Risk of bias in included studies

**Fig. 2:**
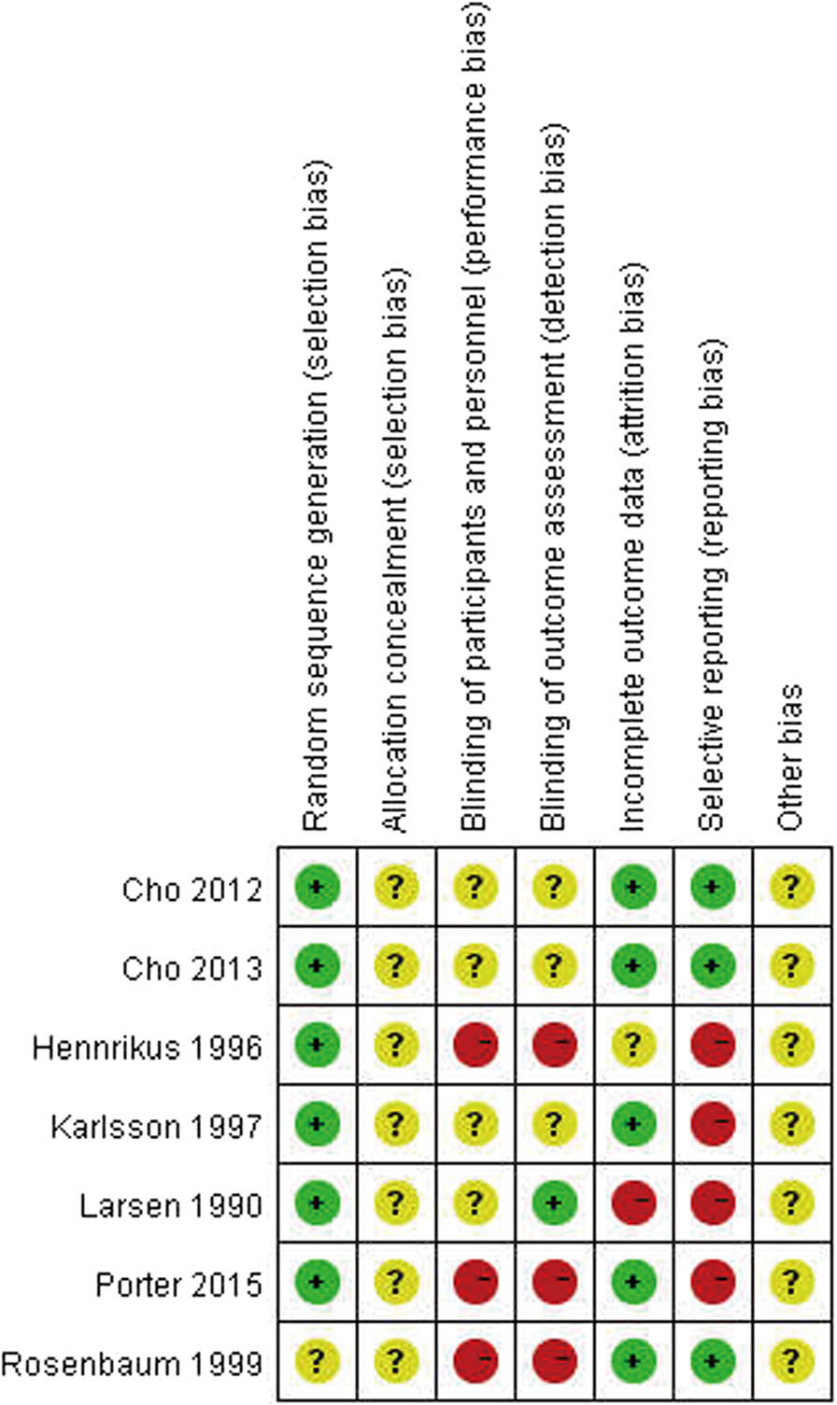
Risk of bias summary: review authors’ judgments about each risk of bias item for each included study

**Fig. 3:**
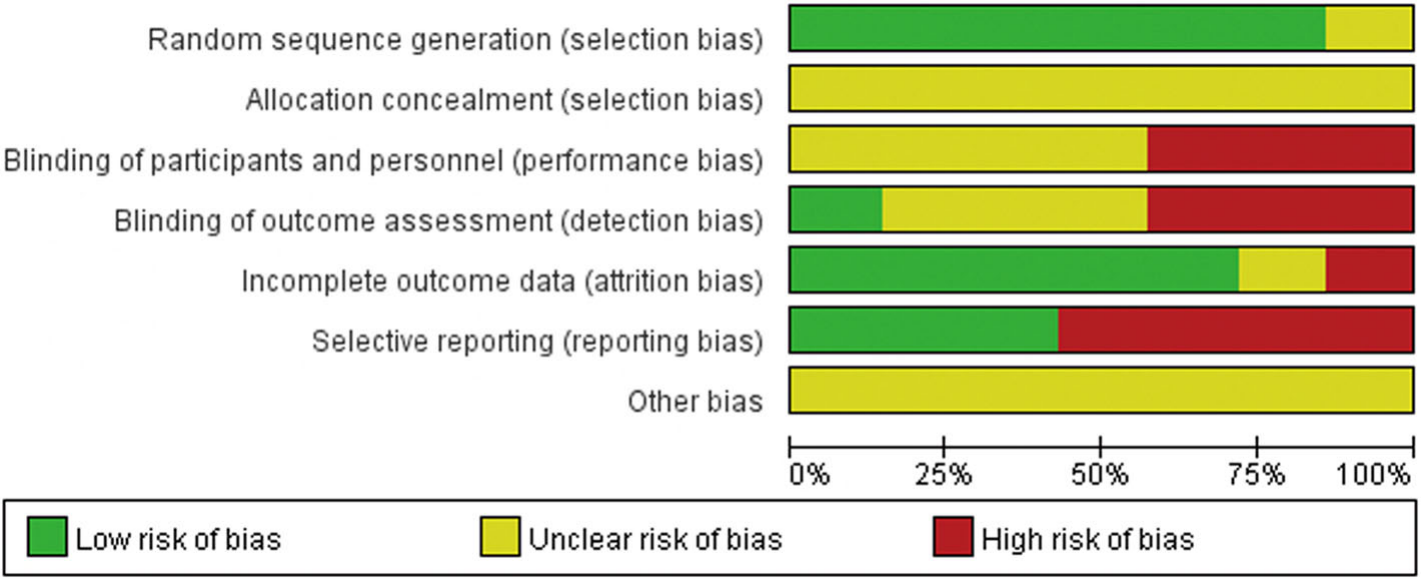
Risk of bias graph: review authors’ judgments about each risk of bias item presented as percentages across all included studies

See the “Risk of bias” tables in characteristics of included studies (Additional files 2, 3, 4, 5, 6, 7, and 8) and Figs. 2 and 3.

#### Allocation (selection bias)

Generation of the allocation sequence was considered of low risk of bias in six trials [63–68] and unclear risk in Rosenbaum et al. [69], the allocation of which was stratified randomized but no further details provided. Comparability of the groups was good and well described in three studies [64, 66, 69]. However, adequate random sequence generation together with adequate allocation concealment could not be confirmed in any of the trails. The other four studies provided no details of comparability.

#### Blinding (performance bias and detection bias)

In Larsen [67], it was well described that radiographic evaluation at the follow-up was blinded; none of the other studies provided information about blinding. We acknowledge blinding can be difficult in the comparison of ligament reconstruction and ligament repairment due to the additional operation incision in Evans/CS/LARS procedure, and thus, Hennrikus et al. [65], Porter et al. [68], and Rosenbaum et al. [69] were judged at being at high risk of both performance and detection bias.

#### Incomplete outcome data (attrition bias)

In three studies [66, 68, 69], there were no participants lost to follow-up. In Hennrikus et al. [65], it was unclear if there were participants lost to follow-up. In Larsen [67], prior to surgery, participants were randomized to one of the two treatment groups (static or dynamic tenodesis), but during the operation, 17 individuals in the dynamic repair group were excluded and not included in the analyses because the procedure was not feasible. In Cho et al. [63] and Cho et al. [64], it was assumed a 20% withdrawal rate pre-operation and analyzed data when each group had enough eligible patients.

#### Selective reporting (reporting bias)

Three studies [63, 64, 69] reported clearly specified outcome measures. We rated the other four studies [65–68] as at high risk of selective reporting bias due to lack of definition of the outcome measures collected in the methods section.

#### Other potential sources of bias

We rated all seven trials as at unclear risk of other bias due to lack of information to make judgments.

### Effects of interventions

#### Non-anatomic reconstruction (static tenodesis) versus non-anatomic reconstruction (dynamic tenodesis)

**Fig. 4:**
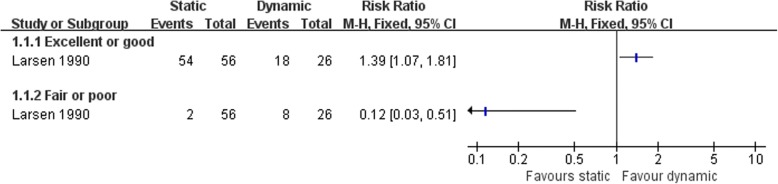
Forest plot of comparison: 1 non-anatomic reconstruction (static tenodesis) versus non-anatomic reconstruction (dynamic tenodesis), outcome: 1.1 satisfaction at 25 months

**Fig. 5:**
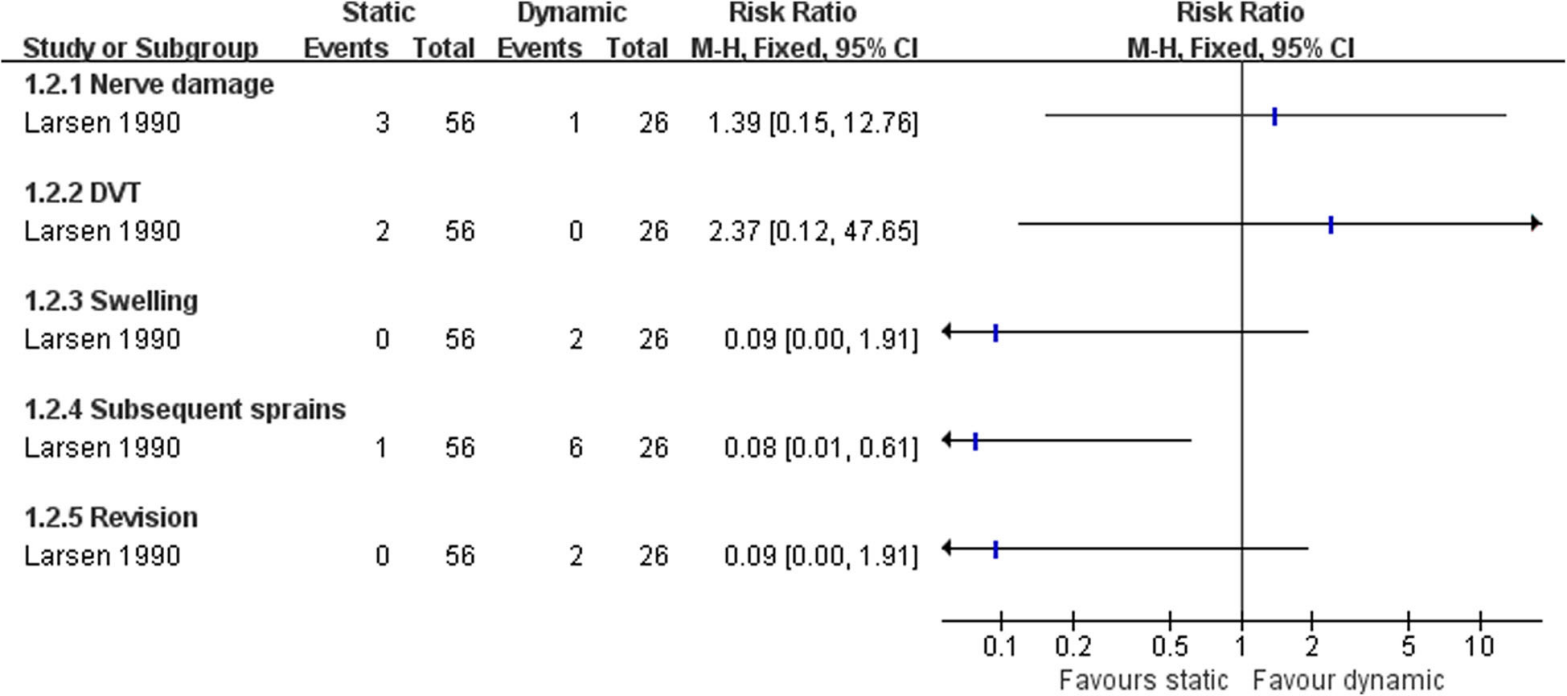
Forest plot of comparison: 1 non-anatomic reconstruction (static tenodesis) versus non-anatomic reconstruction (dynamic tenodesis), outcome: 1.2 complications

Larsen [67] compared the clinical status after two kind of non-anatomic reconstruction procedures, the static and dynamic tenodesis. More patients were satisfied with the static tenodesis than dynamic procedure (Fig. 4). The dynamic tenodesis was associated with more subsequent sprains (Fig. 5). There was no strong evidence to show differences between the two groups in the numbers of participants with other complications (Fig. 5).

#### Non-anatomic reconstruction versus anatomic repairment

**Fig. 6:**
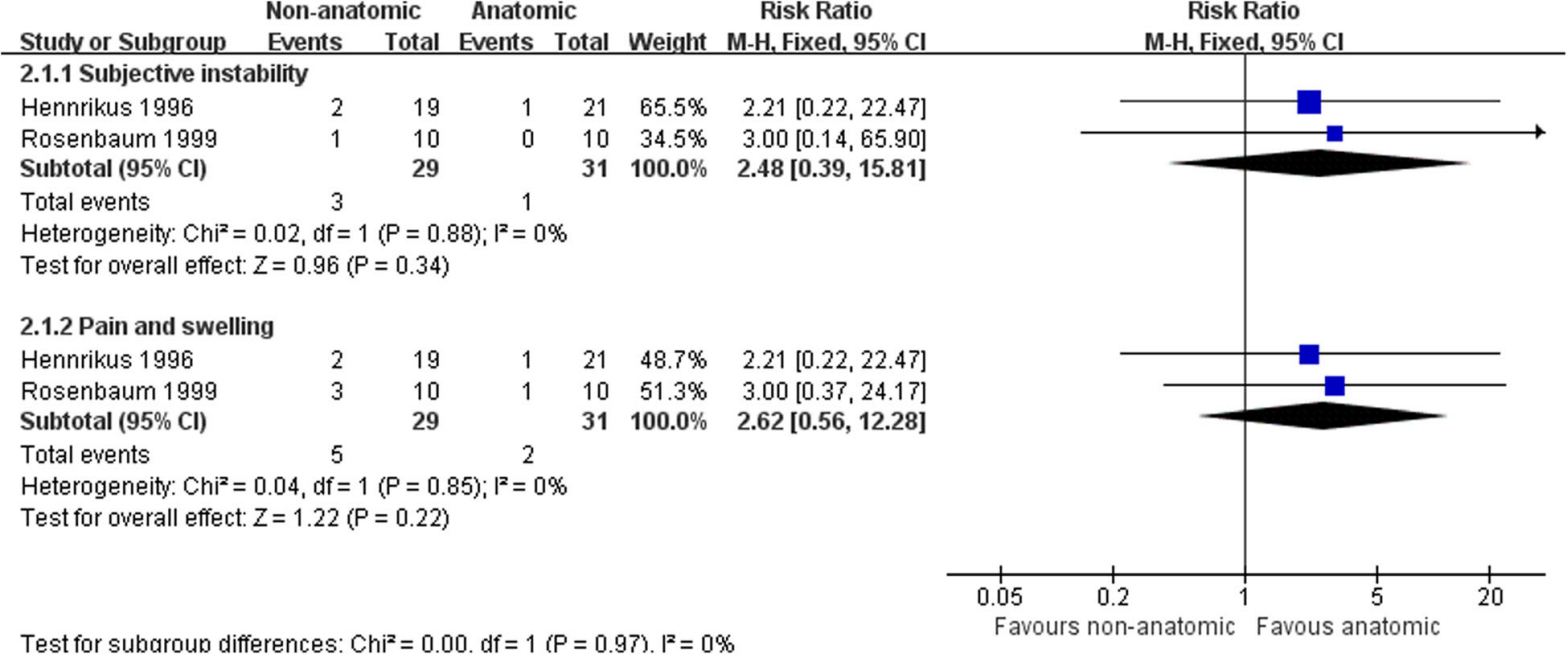
Forest plot of comparison: 2 non-anatomic reconstruction (Evans/CS) versus anatomic repairment (MB), outcome: 2.1 subjective instability, pain, and swelling

**Fig. 7:**
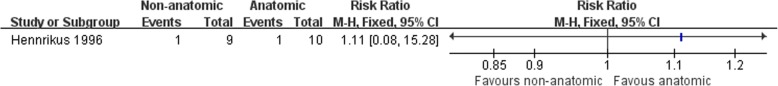
Forest plot of comparison: 2 non-anatomic reconstruction (Evans/CS) versus anatomic repairment (MB), outcome: 2.2 radiographic instability

**Fig. 8:**
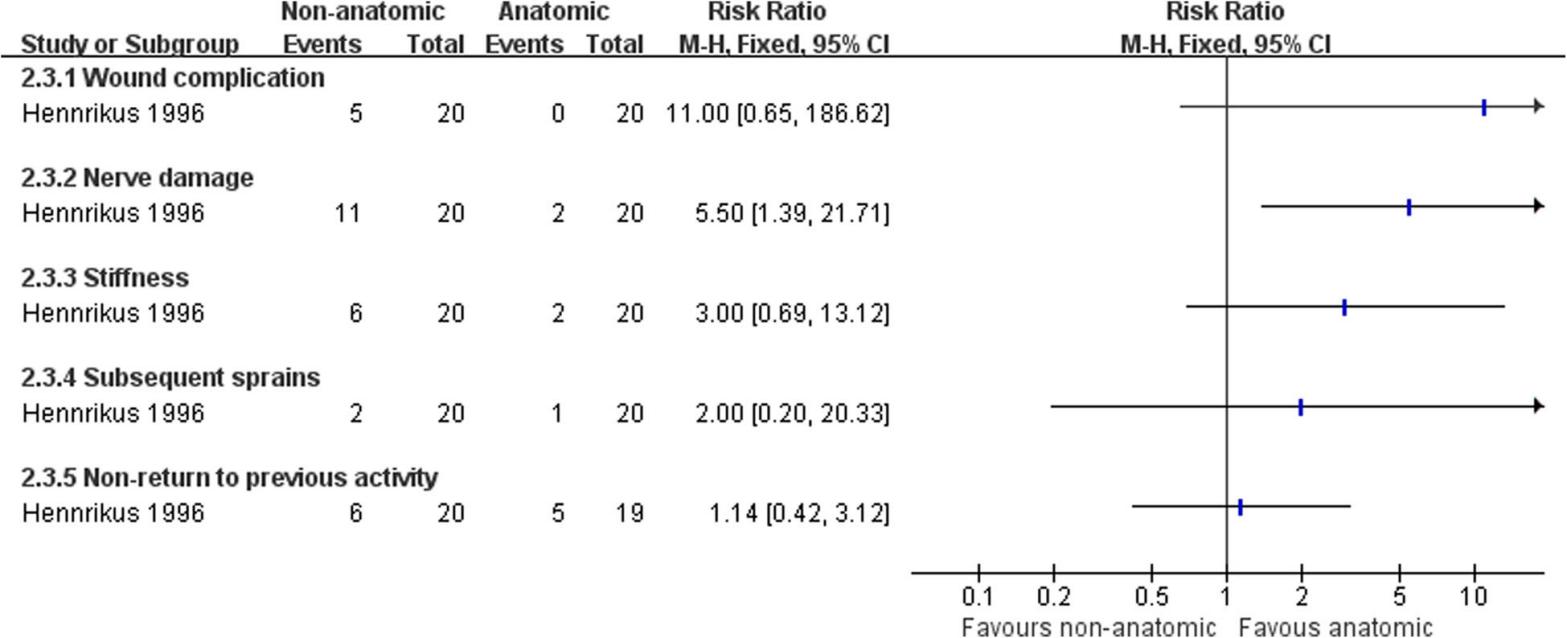
Forest plot of comparison: 2 non-anatomic reconstruction (Evans/CS) versus anatomic repairment (MB), outcome: 2.3 complications

**Fig. 9:**
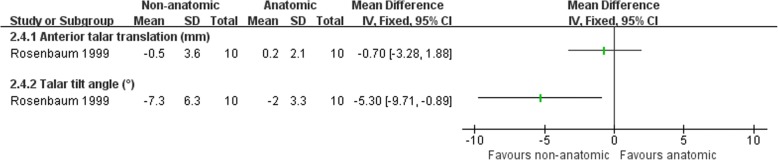
Forest plot of comparison: 2 non-anatomic reconstruction (Evans/CS) versus anatomic repairment (MB), outcome: 2.4 reduction in measures of radiographic ligament laxity

Hennrikus et al. [65] compared the CS procedure with a MB repairment, and Rosenbaum et al. [69] compared a modified Evans procedure with a MB repairment. There was no clear evidence to show differences between the two procedures in subjective instability (3/29 versus 1/31; RR 2.48, 95% CI 0.39 to 15.81) or pain and swelling at the follow-up (5/29 versus 2/31; RR 2.62, 95% CI 0.56 to 12.28) (Fig. 6). Hennrikus et al. [65] found a higher rate of nerve damage in the non-anatomic reconstruction (11/20 versus 2/20; RR 5.50, 95% CI 1.39 to 21.71) (Fig. 8). There was no difference between the two procedures in the other complications and radiographic instability (Figs. 7 and 8). There was strong evidence that the non-anatomic reconstruction group have a greater reduction of the talar tilt angle (MD − 5.3°, 95% CI − 9.71 to − 0.89) (Fig. 9). However, there was no clear evidence to show difference in reduction of anterior talar translation (MD − 0.70 mm, 95% CI − 3.28 to 1.88).

#### Anatomic repairment (transosseous suture) versus anatomic repairment(imbrication)

**Fig. 10:**
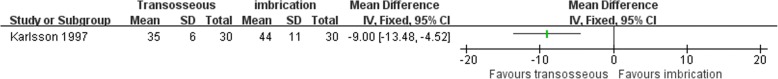
Forest plot of comparison: 3 anatomic repairment (transosseous suture, MB) versus anatomic repairment (imbrication, MB), outcome: 3.1 operating time (minutes)

**Fig. 11:**
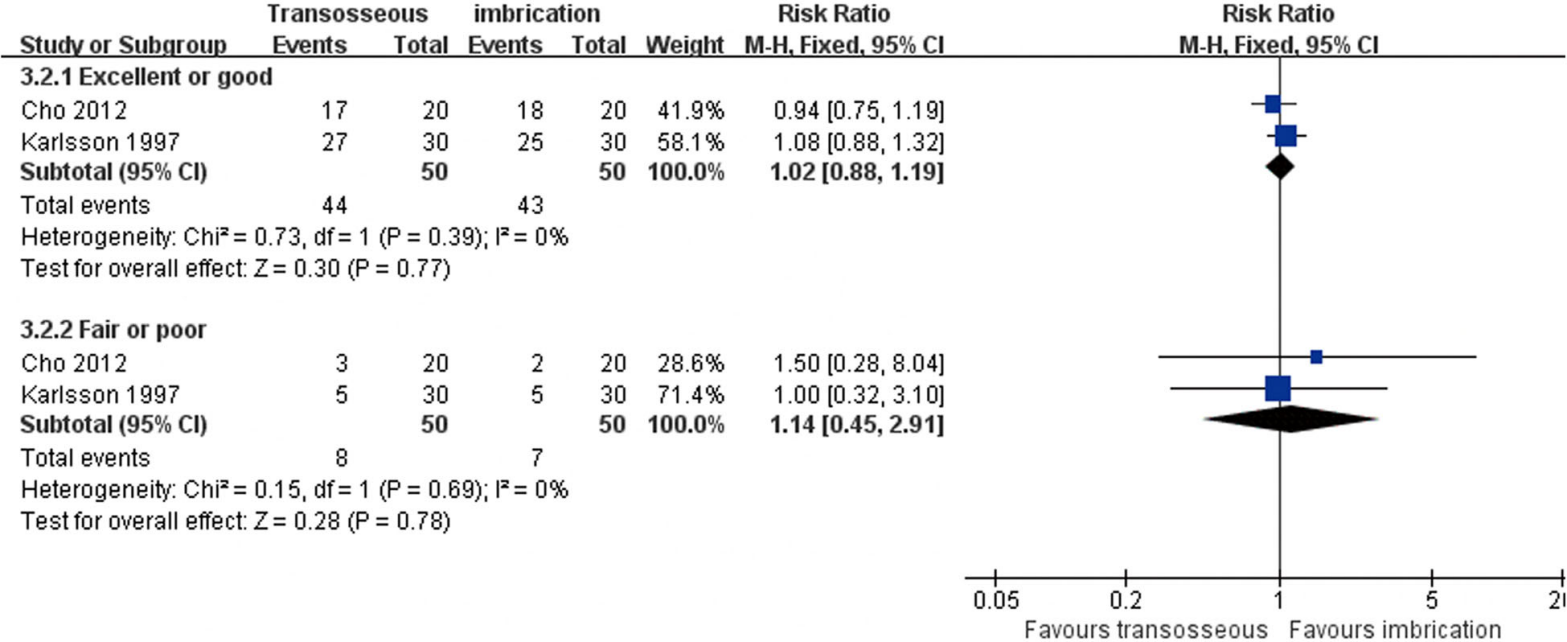
Forest plot of comparison: 3 anatomic repairment (transosseous suture, MB) versus anatomic repairment (imbrication, MB), outcome: 3.2 satisfaction at > 24 months

**Fig. 12:**
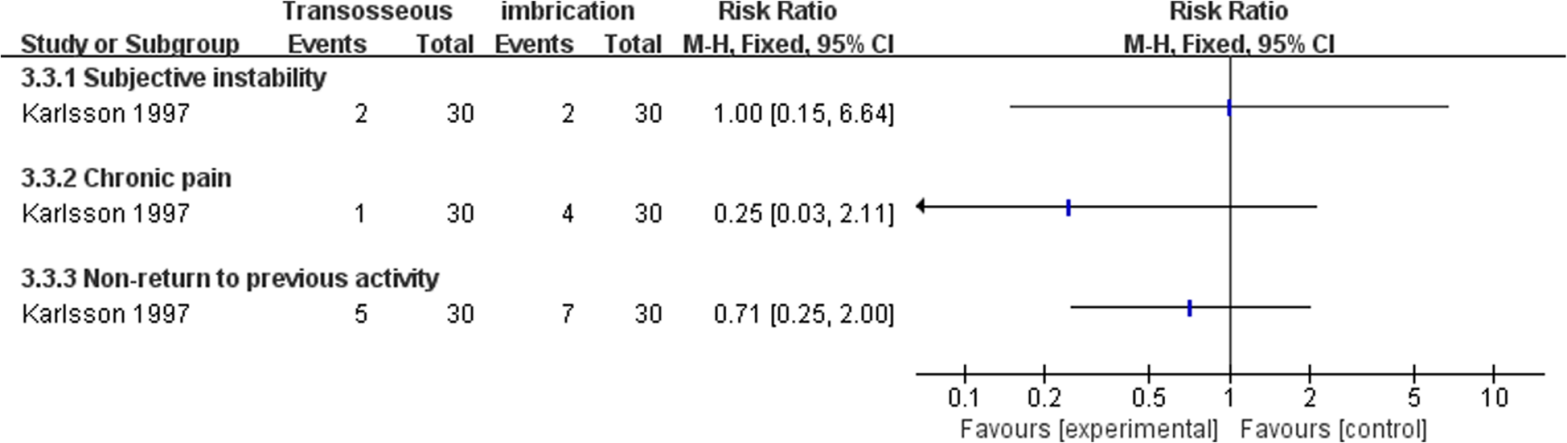
Forest plot of comparison: 3 anatomic repairment (transosseous suture, MB) versus anatomic repairment (imbrication, MB), outcome: 3.3 subjective instability, pain, and activity

**Fig. 13:**
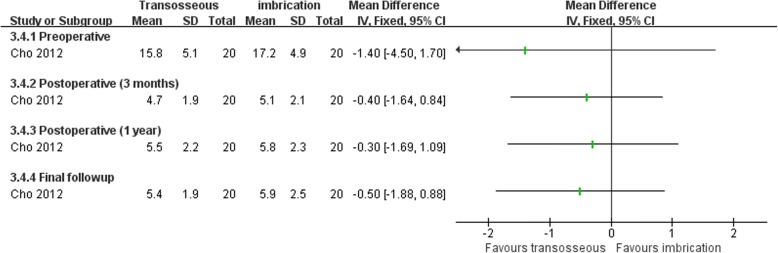
Forest plot of comparison: 3 anatomic repairment (transosseous suture, MB) versus anatomic repairment (imbrication, MB), outcome: 3.4 talar tilt angle (degrees)

**Fig. 14:**
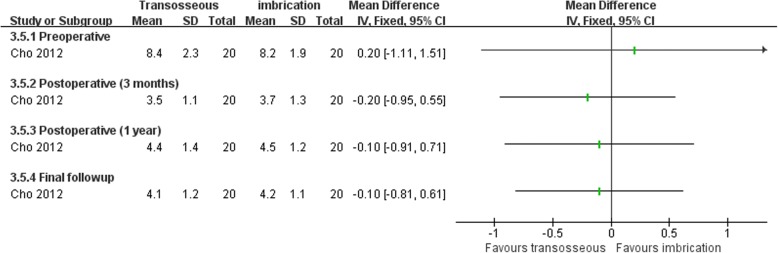
Forest plot of comparison: 3 anatomic repairment (transosseous suture, MB) versus anatomic repairment (imbrication, MB), outcome: 3.5 anterior talar translation (millimeters)

**Fig. 15:**
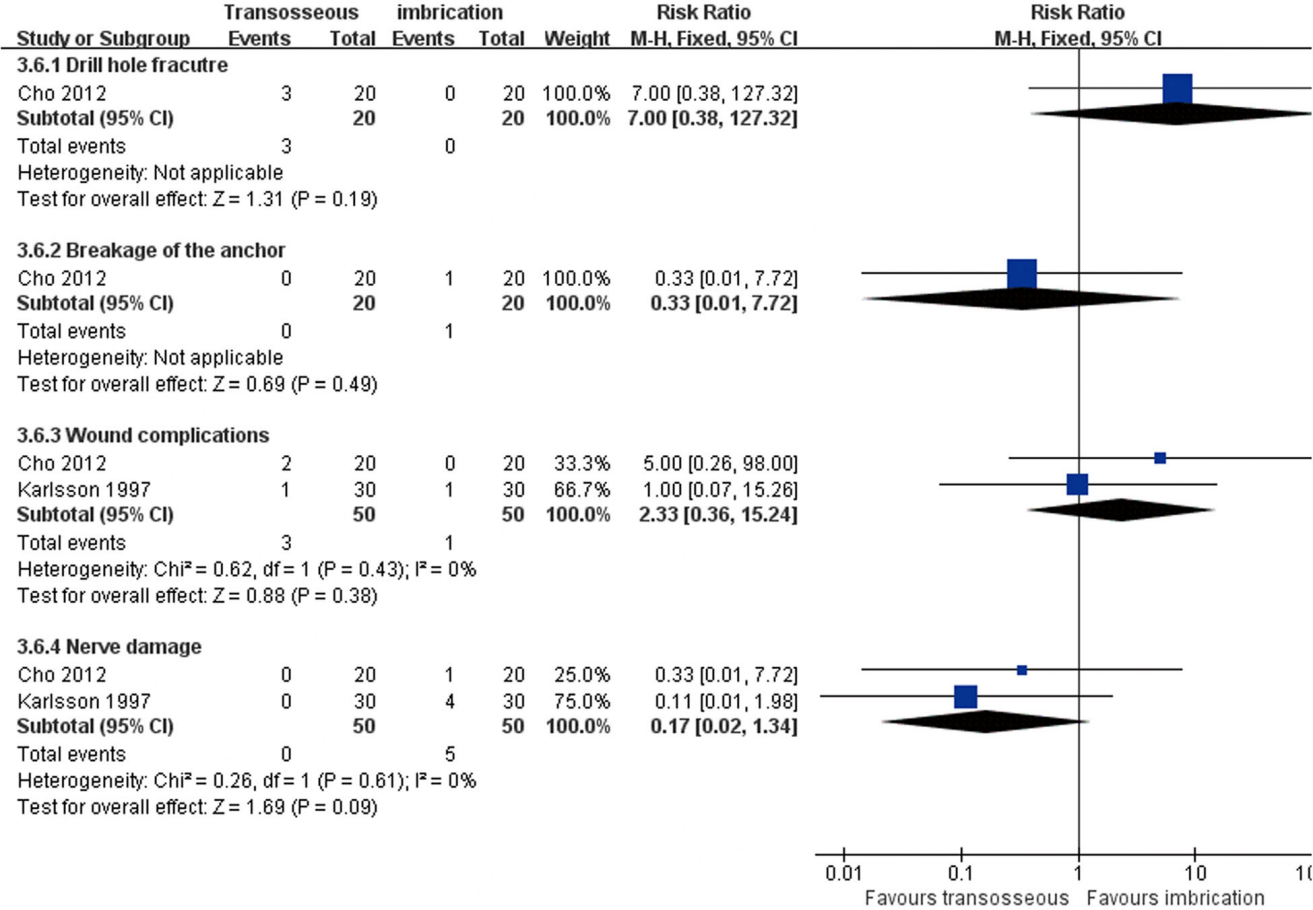
Forest plot of comparison: 3 anatomic repairment (transosseous suture, MB) versus anatomic repairment (imbrication, MB), outcome: 3.6 complications

Both Karlsson et al. [66] and Cho et al. [63] compared transosseous suture and imbrication with inferior extensor retinaculum reinforcement. Karlsson et al. [66] reported that the mean operation time was significantly longer in the imbrication group (Fig. 10). In Cho et al. [63], the imbrication technique also used a single anchor to fix the ATFL and articular capsule. There was no statistically significant difference between the two operations in clinical satisfaction at more than 2 years of follow-up (Fig. 11). Similar findings of non-significant differences between the two groups applied to subjective instability, chronic pain, non-return to previous activity, anterior talar translation, talar tilt angle, and complications (Figs. 12, 13, 14, and 15).

#### Anatomic repairment (single anchor, MB) versus anatomic repairment (double anchors, MB)

**Fig. 16:**
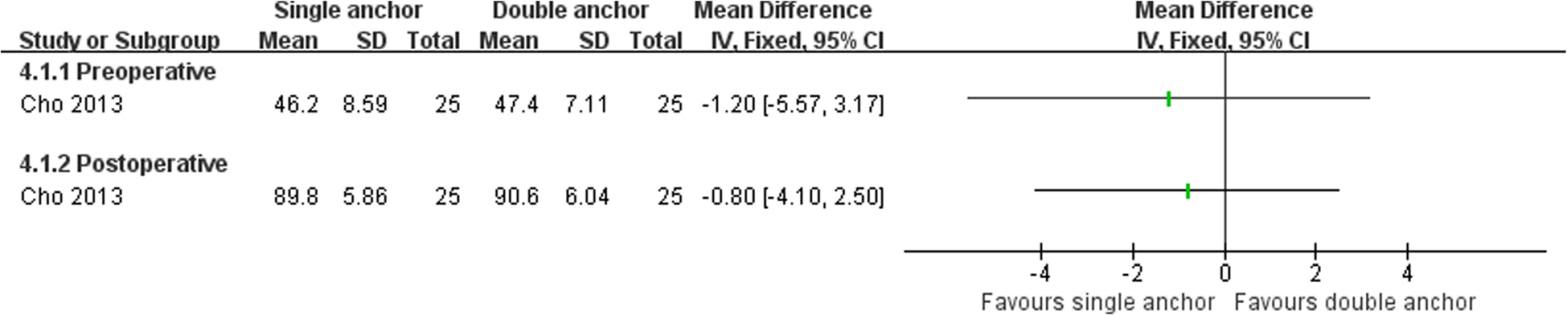
Forest plot of comparison: 4 anatomic repairment (single anchor, MB) versus anatomic repairment (double anchor, MB), outcome:4.1 Karlsson score

**Fig. 17:**
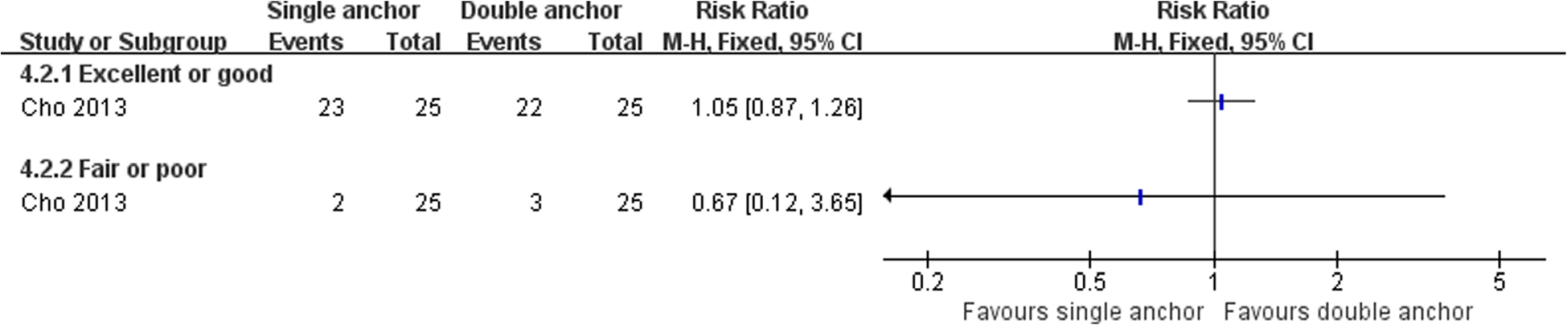
Forest plot of comparison: 4 anatomic repairment (single anchor, MB) versus anatomic repairment (double anchor, MB), outcome: 4.2 satisfaction at > 24 months

**Fig. 18:**
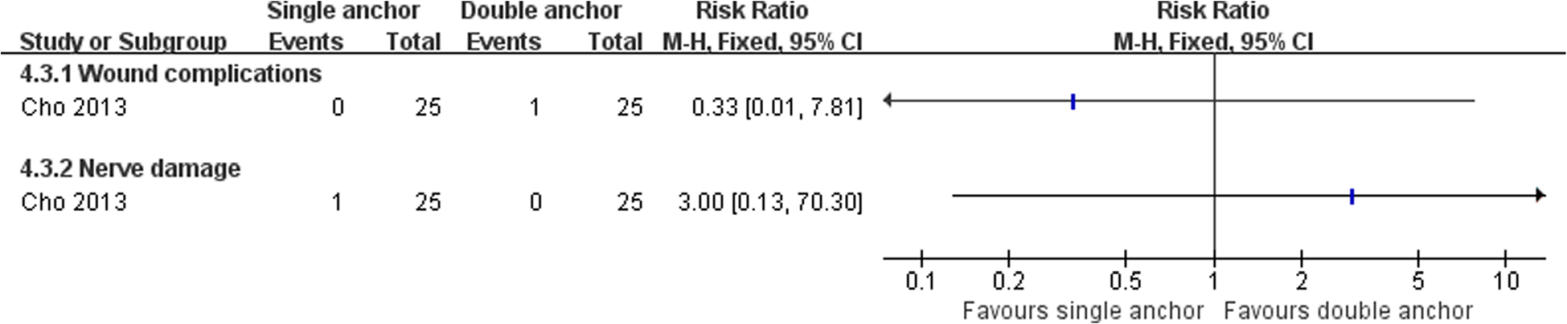
Forest plot of comparison: 4 anatomic repairment (single anchor, MB) versus anatomic repairment (double anchor, MB), outcome: 4.3 complications

**Fig. 19:**
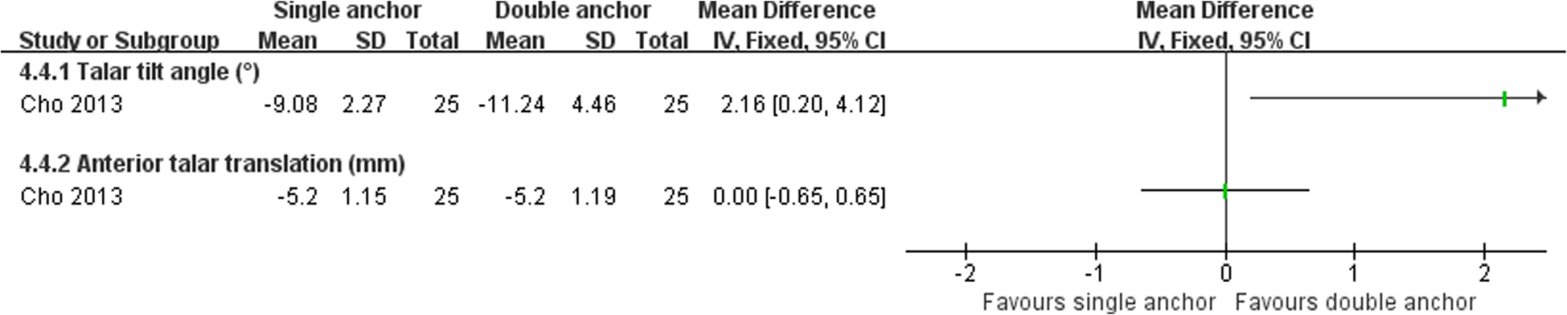
Forest plot of comparison: 4 anatomic repairment (single anchor, MB) versus anatomic repairment (double anchor, MB), outcome: 4.4 reduction in measures of radiographic ligament laxity

Cho et al. [64] compared the clinical outcomes of the MB procedure using single and double suture anchors for chronic lateral ankle instability. There was no statistically significant difference between the two operations in Karlsson score, clinical satisfaction, and postoperative complications (Figs. 16, 17, and 18). The talar tilt angle and anterior talar translation on stress radiographs using the Telos device had improved significantly in the two groups. The double anchor technique was superior with respect to the reduction in talar tilt angle (Fig. 19).

#### Anatomic reconstruction (LARS) versus anatomic repairment (MB)

**Fig. 20:**
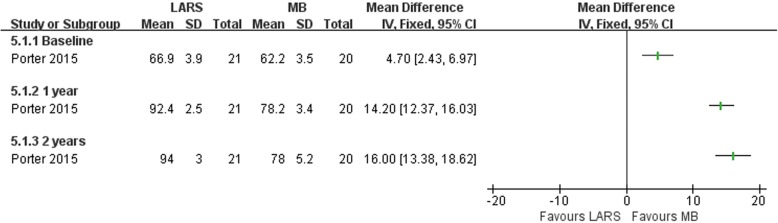
Forest plot of comparison: 5 anatomic reconstruction (LARS) versus anatomic repairment (MB), outcome: 5.1 FAOS. LARS ligament advanced reinforcement system, MB modified Brostrom procedure

**Fig. 21:**
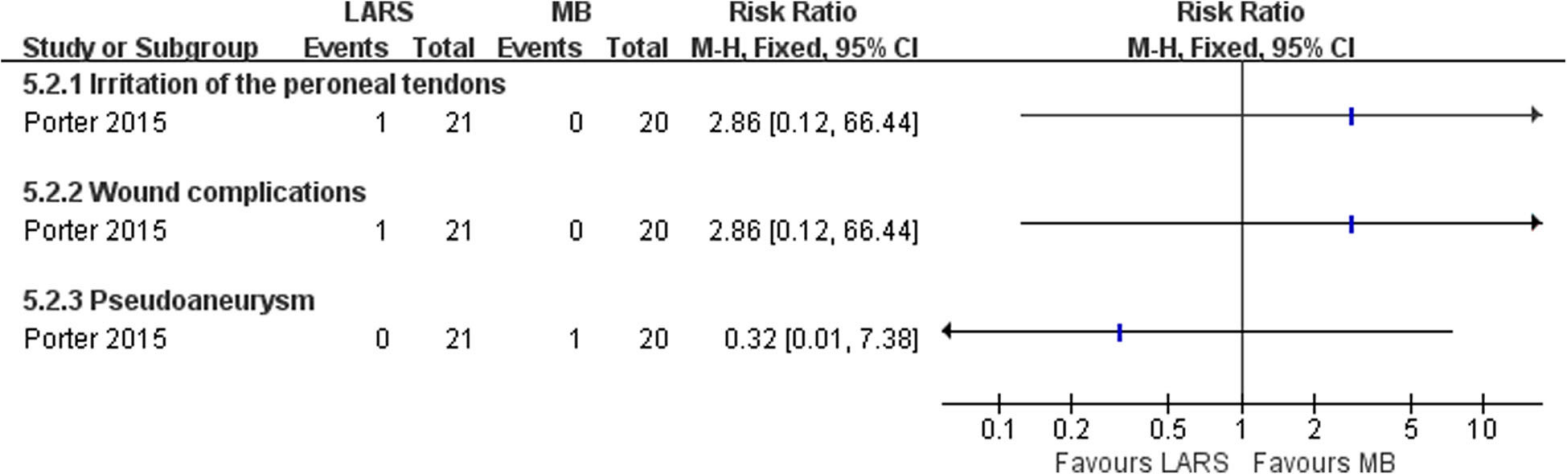
Forest plot of comparison: 5 anatomic reconstruction (LARS) versus anatomic repairment (MB), outcome: 5.2 complications. LARS ligament advanced reinforcement system, MB modified Brostrom procedure

Porter et al. [68] compared an anatomic reconstruction procedure (LARS) with a MB procedure. Forty-one patients took part in the study, 21 were randomized to the LARS group and 20 to the MB group. There was strong evidence that the LARS group had a better improvement in the total FAOS at both 1 year and 2 years post-surgery (Fig. 20), while there was no clear evidence to show difference between the two operations in postoperative complications (Fig. 21).

## Discussion

To date, many surgical procedures have been applied to manage chronic ankle instability, a testament to the complexity of the current status. These procedures and their modifications fall into three categories: non-anatomic reconstruction, anatomic repairment, and anatomic reconstruction. Choosing a suitable surgical procedure is a complex question that has many arguments in the theory and clinical practice. This meta-analysis performed an extensive review of the literature systematically and compared different surgical techniques for management of chronic lateral ankle instability.

### Summary of main results

The seven included studies fell in five clearly distinct groups comprising one study comparing different non-anatomic reconstruction procedures, three studies comparing different anatomic repairment techniques, two studies comparing non-anatomic reconstruction with anatomic repairment, and one study comparing anatomic reconstruction with anatomic repairment for CAI. There were only limited opportunities for pooling of data and few statistically significant differences in clinical outcomes between groups.

Larsen [67] comparing two different kinds of non-anatomic reconstruction procedures (dynamic and static tenodesis) found two outcomes favoring static tenodesis: better clinical satisfaction and fewer subsequent sprains.

Two studies [65, 69] have compared non-anatomic reconstruction versus anatomic repairment. In Hennrikus et al. [65], nerve injury was more frequent in non-anatomic reconstruction group. Rosenbaum et al. [69] reported that radiological measurement of ankle joint relaxation showed that non-anatomic reconstruction provided higher reduction of talar tilt angle.

Analysis of data from two researches [63, 66], comparing two anatomic repairment surgical procedures, showed no significant difference in any clinical outcome at follow-up, but Karlsson et al. [66] reported that the average operation time was significantly longer in the imbrication group.

One randomized study [64], comparing two different anatomic repairment techniques, found that the double anchor technique was superior with respect to the postoperative talar tilt than single anchor technique.

The other one study [68] compared an anatomic reconstruction procedure with a MB technique. Primary repair combined with LARS results in better patient-scored clinical outcome, at 2 years post-surgery, than the MB procedure.

### Overall completeness and applicability of evidence

The search and selection criteria led to the inclusion of seven studies, which are somewhat heterogeneous. In all studies, interventions were described adequately, but the specific concrete operation steps of surgical protocols were different. In the section of non-anatomic reconstruction versus anatomic repairment, Hennrikus et al. [65] used CS procedure and Rosenbaum et al. [69] used a modified Evans procedure. In the section of transosseous suture anatomic repairment versus imbrication anatomic repairment, Cho et al. [63] used a single anchor to fix ATFL and articular capsule, while Karlsson et al. [66] did not. Inclusion criteria were adequately described in all studies. In three studies, the exclusion criteria were clearly described as well [63, 65, 68]. The other four studies did not describe exclusion criteria or only mentioned a few criteria that did not exclude all conditions that could influence outcome.

### Quality of the evidence

Limitations in the conduct, design, and reporting of the trials resulted in judgements of high risk or unclear of selection bias, attrition bias, detection bias, and reporting bias in one or more trials.

### Potential biases in the review process

Although the search strategy was comprehensive and our methods of study selection were thorough, publication bias, study selection bias, and study identification bias can never completely be excluded.

### Agreements and disagreements with other studies or reviews

Two systematic reviews evaluating surgical treatment techniques for CAI have been published [70, 71]. They included non-operative treatment and post-surgical rehabilitation studies as well. This review also includes three studies published after 2011.

## Conclusions

Limited by the unclear to high risk of bias, few high-quality studies, and clinical heterogeneity, this review does not provide strong evidence on which to base practice. Based on the evidence, non-anatomic reconstruction abnormally increased inversion stiffness at the subtalar level as compare with anatomic repairment; this kind of procedure should be avoided to be the first-line surgical choice. Anatomic repairment procedure and anatomic reconstruction could acquire good clinical results; they are the appropriate first-line consideration for patients with chronic lateral ankle ligament laxity requiring surgical treatment. There is a need for high-quality randomized controlled trials evaluating the surgical treatment of chronic lateral ankle instability. Minimize bias and sufficient follow-up period are important for all trials.

## Additional files

Additional file 1: Search strategies (DOCX 14 kb)

Additional file 2: Characteristics of included studies 1 (PDF 88 kb)

Additional file 3: Characteristics of included studies 2 (PDF 91 kb)

Additional file 4: Characteristics of included studies 3 (PDF 89 kb)

Additional file 5: Characteristics of included studies 4 (PDF 93 kb)

Additional file 6: Characteristics of included studies 5 (PDF 91 kb)

Additional file 7: Characteristics of included studies 6 (PDF 91 kb)

Additional file 8: Characteristics of included studies 7 (PDF 85 kb)

Additional file 9: Characteristics of excluded studies (PDF 90 kb)

## Abbreviations

ATFL: Anterior talofibular ligament
CAI: Chronic ankle instability
CFL: Calcaneofibular ligament
CS: Chrisman-Snook
FAOS: Foot and ankle outcome score
LARS: Ligament advanced reinforcement system
MB: Modified Brostrom procedure
PTFL: Posterior talofibular ligament
