# Supplementary material for: Surgical management of chronic lateral ankle instability: a meta-analysis
Source: J Orthop Surg Res. 2018 Jun 25;13:159. doi: 10.1186/s13018-018-0870-6 (PMC6019311; doi:10.1186/s13018-018-0870-6)
Supplement: Supplementary file 1 — Search strategies (DOCX 14 kb) [file 13018_2018_870_MOESM1_ESM.docx]

**Search strategies for Cochrane**

ID Search

#1 MeSH descriptor: [Ankle] explode all trees

#2 MeSH descriptor: [Ankle Injuries] explode all trees

#3 MeSH descriptor: [Ankle Joint] explode all trees

#4 MeSH descriptor: [Lateral Ligament, Ankle] explode all trees

#5 MeSH descriptor: [Ligaments] explode all trees

#6 ankle* near/5 (injur* or joint* or ligament* or sprain* or strain* or inversion*):ti,ab,kw (Word variations have been searched)

#7 ankle:ti,ab,kw (Word variations have been searched)

#8 (#1 or #7) and #5

#9 #2 or #3 or #4 or #6 or #8

#10 MeSH descriptor: [Joint Instability] explode all trees

#11 MeSH descriptor: [Chronic Disease] explode all trees

#12 instability or unstable or lax* or recurrent or chronic*:ti,ab,kw (Word variations have been searched)

#13 #10 or #11 or #12

#14 #9 and #13

**Search strategies for MEDLINE**

Search Query

#24 Search (((((((lateral ligament, ankle[MeSH Terms]) OR ankle injuries[MeSH Terms]) OR ankle joint[MeSH Terms]) OR ((((ankle) OR ankle[MeSH Terms])) AND ((((((injur*) OR joint*) OR ligament*) OR sprain*) OR starin*) OR inversion*)))) AND (((((((sprains and strains[MeSH Terms]))) OR joint instability[MeSH Terms]) OR ligament[MeSH Terms]) OR chronic disease[MeSH Terms]) OR (((((instability) OR unstable) OR lax*) OR recurrent) OR chronic*)))) AND (((((((randomized controlled trial[Publication Type]) OR controlled clinical trial[Publication Type]) OR randomized[Title/Abstract]) OR placebo[Title/Abstract]) OR clinical trials as topic[MeSH Major Topic]) OR randomly[Title/Abstract]) OR trial[Title])

#23 Search ((((((randomized controlled trial[Publication Type]) OR controlled clinical trial[Publication Type]) OR randomized[Title/Abstract]) OR placebo[Title/Abstract]) OR clinical trials as topic[MeSH Major Topic]) OR randomly[Title/Abstract]) OR trial[Title]

#22 Search trial[Title]

#21 Search randomly[Title/Abstract]

#20 Search clinical trials as topic[MeSH Major Topic]

#19 Search placebo[Title/Abstract]

#18 Search randomized[Title/Abstract]

#17 Search controlled clinical trial[Publication Type]

#16 Search randomized controlled trial[Publication Type]

#15 Search (((((lateral ligament, ankle[MeSH Terms]) OR ankle injuries[MeSH Terms]) OR ankle joint[MeSH Terms]) OR ((((ankle) OR ankle[MeSH Terms])) AND ((((((injur*) OR joint*) OR ligament*) OR sprain*) OR starin*) OR inversion*)))) AND (((((((sprains and strains[MeSH Terms]))) OR joint instability[MeSH Terms]) OR ligament[MeSH Terms]) OR chronic disease[MeSH Terms]) OR (((((instability) OR unstable) OR lax*) OR recurrent) OR chronic*))

#14 Search ((((((sprains and strains[MeSH Terms]))) OR joint instability[MeSH Terms]) OR ligament[MeSH Terms]) OR chronic disease[MeSH Terms]) OR (((((instability) OR unstable) OR lax*) OR recurrent) OR chronic*)

#13 Search ((((instability) OR unstable) OR lax*) OR recurrent) OR chronic*

#12 Search chronic disease[MeSH Terms]

#11 Search ligaments, articular[MeSH Terms]

#10 Search ligament[MeSH Terms]

#9 Search joint instability[MeSH Terms]

#8 Search (sprains and strains[MeSH Terms])

#7 Search (((lateral ligament, ankle[MeSH Terms]) OR ankle injuries[MeSH Terms]) OR ankle joint[MeSH Terms]) OR ((((ankle) OR ankle[MeSH Terms])) AND ((((((injur*) OR joint*) OR ligament*) OR sprain*) OR starin*) OR inversion*))

#6 Search (((ankle) OR ankle[MeSH Terms])) AND ((((((injur*) OR joint*) OR ligament*) OR sprain*) OR starin*) OR inversion*)

#5 Search (((((injur*) OR joint*) OR ligament*) OR sprain*) OR starin*) OR inversion*

#4 Search (ankle) OR ankle[MeSH Terms]

#3 Search ankle joint[MeSH Terms]

#2 Search ankle injuries[MeSH Terms]

#1 Search lateral ligament, ankle[MeSH Terms]

**Search strategies for EMBASE**

No. Query

#19 'chronic disease' OR 'joint laxity' OR instability OR unstable OR lax* OR recurrent OR chronic* AND ('ankle lateral ligament' OR 'ankle instability' OR 'ankle injury' OR 'ankle sprain' OR ankle* NEAR/5 (injur* OR sprain* OR joint* OR ligament* OR strain* OR inversion*) OR ('ligament injury' AND ankle*)) AND ([cochrane review]/lim OR [systematic review]/lim OR [meta analysis]/lim OR [controlled clinical trial]/lim OR [randomized controlled trial]/lim)

#18 'chronic disease' OR 'joint laxity' OR instability OR unstable OR lax* OR recurrent OR chronic* AND ('ankle lateral ligament' OR 'ankle instability' OR 'ankle injury' OR 'ankle sprain' OR ankle* NEAR/5 (injur* OR sprain* OR joint* OR ligament* OR strain* OR inversion*) OR ('ligament injury' AND ankle*))

#17 'ankle lateral ligament' OR 'ankle instability' OR 'ankle injury' OR 'ankle sprain' OR ankle* NEAR/5 (injur* OR sprain* OR joint* OR ligament* OR strain* OR inversion*) OR ('ligament injury' AND ankle*)

#16 'chronic disease' OR 'joint laxity' OR instability OR unstable OR lax* OR recurrent OR chronic*

#15 instability OR unstable OR lax* OR recurrent OR chronic*

#14 'joint laxity'

#13 'chronic disease'

#10 'ligament injury' AND ankle*

#9 ankle*

#8 'ligament injury'

#6 'ankle lateral ligament' OR 'ankle instability' OR 'ankle injury' OR 'ankle sprain' OR ankle* NEAR/5 (injur* OR sprain* OR joint* OR ligament* OR strain* OR inversion*)

#5 ankle* NEAR/5 (injur* OR sprain* OR joint* OR ligament* OR strain* OR inversion*)

#4 'ankle sprain'

#3 'ankle injury'

#2 'ankle instability'

#1 'ankle lateral ligament'
