# Supplementary material for: Surgical management of chronic lateral ankle instability: a meta-analysis
Source: J Orthop Surg Res. 2018 Jun 25;13:159. doi: 10.1186/s13018-018-0870-6 (PMC6019311; doi:10.1186/s13018-018-0870-6)
Supplement: Supplementary file 2 — Characteristics of included studies 1 (PDF 88 kb) [file 13018_2018_870_MOESM2_ESM.pdf]

Characteristics of studies

Characteristics of included studies

Cho 2012

**Methods**	Location: Chungbuk National University Hospital<br>Design: Prospective Randomised Trial<br>Method of randomisation: Table of random numbers<br>Assessor blinding: Not mentioned<br>Study period: Not mentioned<br>Follow-up: More than 2 years<br>Intention-to-treat: Assumed a 20% dropout-rate preoperation and analyzed data when each group had 20 eligible patients.
**Participants**	Transosseous suture group: 12 males and 8 females, Suture anchor group: 11 males and 9 females<br>Mean age of the patients: 33.9 (range, 21 to 42) years in the transosseous suture group; 30.7 (range, 15 to 44) years in the suture anchor group.<br>Inclusion criteria (1+2+3/4):<br>(1) patients who complained of subjective instability of the ankle joint in whom repeated sprain injuries for more than 6 months and pain were confirmed;<br>(2) patients with marked ankle instability confirmed by the anterior drawer test compared with the contralateral ankle and tenderness involving the lateral ligaments of the ankle confirmed on physical examination;<br>(3) patients with a talar tilt angle exceeding 10 degrees or a discrepancy of more than 5 degrees compared with the non-affected side on stress radiography;<br>(4) patients with an anterior talar translation exceeding 10 mm or a discrepancy more than 3 mm compared with the nonaffected side.
**Interventions**	Two methods of ankle ligament reconstruction:<br>(1) Suture Anchor for the Modified Brostrom Procedure<br>(2) Transosseous Suture for the Modified Brostrom Procedure
**Outcomes**	(1) Karlsson score<br>(2) Sefton grading system<br>(3) Anterior talar translation and talar tilt angle (preoperative and postoperative)<br>(4) Intraoperative and postoperative complications: drill hole fracture, breakage of the anchor, wound complications and nerve damage
**Notes**	

Risk of bias table

Bias	Authors' judgement	Support for judgement
Random sequence generation (selection bias)	Low risk	Patients were randomly assigned based on a table of random numbers
Allocation concealment (selection bias)	Unclear risk	Allocation concealment not mentioned
Blinding of participants and personnel (performance bias)	Unclear risk	Blinding not mentioned
Blinding of outcome assessment (detection bias)	Unclear risk	Blinding not mentioned
Incomplete outcome data (attrition bias)	Low risk	Assumed dropout-rate preoperation, probably no data lost
Selective reporting (reporting bias)	Low risk	Outcome measures the same in methods and results sections

Other bias	Unclear risk	There was insufficient information to judge the risk from other sources of bias

Footnotes
