# Supplementary material for: Surgical management of chronic lateral ankle instability: a meta-analysis
Source: J Orthop Surg Res. 2018 Jun 25;13:159. doi: 10.1186/s13018-018-0870-6 (PMC6019311; doi:10.1186/s13018-018-0870-6)
Supplement: Supplementary file 3 — Characteristics of included studies 2 (PDF 91 kb) [file 13018_2018_870_MOESM3_ESM.pdf]

Characteristics of studies

Characteristics of included studies

Cho 2013

**Methods**	Location: Chungbuk National University Hospital<br>Design: Prospective randomized comparison trial<br>Method of randomisation: Table of random numbers<br>Assessor blinding: Not mentioned<br>Study period: Not mentioned<br>Follow-up: More than 2 years<br>Intention-to-treat: Assumed a 20% withdrawal rate preoperation and analyzed data when each group had 25 eligible patients.
**Participants**	Single anchor group: 6 patients had osteochondral lesions of the talus, 5 had anterior soft tissue impingement syndrome, 2 had anterior bony impingement, and 2 had loose bodies<br>Double anchor group: 4 patients had osteochondral lesions of the talus, 8 had anterior soft tissue impingement syndrome, and 1 had loose bodie<br>Inclusion criteria (1+2+3/4):<br>(1) patients who complained of subjective instability of the ankle joint in whom repeated sprain injuries for more than 6 months and pain were confirmed;<br>(2) patients with marked ankle instability confirmed by the anterior drawer test compared with the contralateral ankle and tenderness involving the lateral ligaments of the ankle confirmed on physical examination;<br>(3) patients with a talar tilt angle exceeding 10 degrees or a discrepancy of more than 5 degrees compared with the non-affected side on stress radiography;<br>(4) patients with an anterior talar translation exceeding 10 mm or a discrepancy more than 3 mm compared with the nonaffected side.
**Interventions**	a) Single anchor group: Modified Brostrom procedure with a single Corkscrew for repairment of the anterolateral ankle ligaments<br>b) Double anchor group: Modified Brostrom procedure with 2 Corkscrew for repairment of the anterolateral ankle ligaments<br>Both groups underwent the same post-operative rehabilitation programme<br>Mean length of follow-up: 30.2 (range 24 to 36) months in the single anchor group and 29.8 (range 24 to 34) months in the double anchor group
**Outcomes**	(1) Karlsson score<br>(2) Sefton grading system<br>(3) Anterior talar translation and talar tilt angle (preoperative and postoperative)<br>(4) Postoperative complications: wound complications, nerve damage, ganglion, device breakage / pull-out / migration.
**Notes**	

Risk of bias table

**Bias**	**Authors' judgement**	**Support for judgement**
Random sequence generation (selection bias)	Low risk	Patients were randomly assigned based on a table of random numbers
Allocation concealment (selection bias)	Unclear risk	Allocation concealment not mentioned
Blinding of participants and personnel (performance bias)	Unclear risk	Blinding not mentioned

Blinding of outcome assessment (detection bias)	Unclear risk	Blinding not mentioned
Incomplete outcome data (attrition bias)	Low risk	Assumed withdrawal rate preoperation, probably no data lost
Selective reporting (reporting bias)	Low risk	Outcome measures the same in methods and results sections
Other bias	Unclear risk	There was insufficient information to judge the risk from other sources of bias

Footnotes
