# Supplementary material for: Surgical management of chronic lateral ankle instability: a meta-analysis
Source: J Orthop Surg Res. 2018 Jun 25;13:159. doi: 10.1186/s13018-018-0870-6 (PMC6019311; doi:10.1186/s13018-018-0870-6)
Supplement: Supplementary file 4 — Characteristics of included studies 3 (PDF 89 kb) [file 13018_2018_870_MOESM4_ESM.pdf]

Characteristics of studies

Characteristics of included studies

Hennrikus 1996

**Methods**	Location: Department of Orthopaedic Surgery and Clinical Investigation, Naval Hospital<br>Design: Randomised controlled trial<br>Method of randomisation: Numbered envelopes with random allocated numbers<br>Assessor blinding: Not mentioned<br>Study period: July 1989 to August 1992<br>Follow-up: Mean 29 months, range 6 to 49 months<br>Loss to follow-up: Two patients lost to follow-up for the final evaluation, not analysed
**Participants**	40 participants, 42 ankles, 4 females and 36 males, mean age 26 years (range 19 to 37)<br>Inclusion criteria:<br>(1) skeletal maturity<br>(2) history of significant ankle injury followed by episodes of giving way for at least 6 months<br>(3) positive anterior drawer test on physical examination<br>Exclusion criteria:<br>(1) Generalized ligamentous laxity disorder;<br>(2) Radiographic arthritis or tarsal coalition on radiographs;<br>(3) Previous ankle surgery<br>Loss to follow-up: Two in Chrisman-Snook group
**Interventions**	Two methods of ankle ligament reconstruction:<br>(1) Chrisman-Snook procedure<br>(2) Modified-Brostrom procedure<br>Assigned: 20 (all males) / 20 (male 16, female 4)<br>Analysed: short term outcomes: 20 / 20, long term outcomes: 18 / 20<br>Physical examination and radiographs: 9 / 10
**Outcomes**	(1) Sefton score<br>(2) Residual instability, pain and swelling<br>(3) Radiographic stability: Anterior talar translation and talar tilt<br>(4) Postoperative complications: wound infection, nerve damage, stiffness, subsequent sprains, non-return to previous activity
**Notes**	

Risk of bias table

**Bias**	**Authors' judgement**	**Support for judgement**
Random sequence generation (selection bias)	Low risk	Numbered envelopes with randomly allocated assignments
Allocation concealment (selection bias)	Unclear risk	Numbered envelopes used, further concealment protection not mentioned
Blinding of participants and personnel (performance bias)	High risk	Blinding not mentioned
Blinding of outcome assessment (detection bias)	High risk	Blinding not mentioned
Incomplete outcome data (attrition bias)	Unclear risk	Two patients lost to final follow-up in Chrisman-Snook group. Lost data not mentioned

Selective reporting (reporting bias)	High risk	Not all outcome measures mentioned in the results selection are described in the methods section
Other bias	Unclear risk	There was not sufficient information to judge the risk from other sources of bias.

Footnotes
