# Supplementary material for: Surgical management of chronic lateral ankle instability: a meta-analysis
Source: J Orthop Surg Res. 2018 Jun 25;13:159. doi: 10.1186/s13018-018-0870-6 (PMC6019311; doi:10.1186/s13018-018-0870-6)
Supplement: Supplementary file 5 — Characteristics of included studies 4 (PDF 93 kb) [file 13018_2018_870_MOESM5_ESM.pdf]

Characteristics of studies

Characteristics of included studies

Karlsson 1997

**Methods**	Location: Ostra Hospital, Goteborg, Sweden<br>Design: Prospective randomized study<br>Method of randomisation: Closed envelopes with the group assignment<br>Assessor blinding: Not mentioned<br>Study period: 1989 to 1992<br>Follow-up: Mean 3.1 / 3.3 years, range 2 to 5 years in both groups<br>Intention-to-treat: Complete follow-up
**Participants**	60 participants, 42 men and 18 women, mean age of 24 years (range, 17 to 36)<br>Inclusion criteria:<br>(1) Chronic ankle instability for more than 6 months<br>(2) Pre-operative supervised rehabilitation programme without success<br>(3) Radiographic measurements: difference in anterior talar translation of ⩾3 mm or talar tilt ⩾3° compared with the contralateral side<br>Loss to follow-up: No patients lost.
**Interventions**	(1) Group Ⅰ: Anatomic repairment of the lateral ankle ligaments by transosseous suture, viewed as a type of Modified Brostrome procedure.<br>(2) Group Ⅱ: Anatomic repairment of the lateral ankle ligaments by imbrication and with inferior extensor retinaculum reinforcement, Modified Brostrom procedure.<br>Both groups underwent the same post-operative rehabilitation programme.<br>Assigned: 30/30<br>Analysed: 30/30 (Two patients, one in each group, both with excellent functional results, didn't participate radiologic follow-up examination.)
**Outcomes**	(1) Operation time<br>(2) Karlsson score (excellent: 91-100; good: 81-90; fair: 61-80; poor: <60)<br>(3) Radiographic stability: Anterior talar translation and talar tilt<br>(4) Postoperative complications: wound infection, nerve damage
**Notes**	

Risk of bias table

Bias	Authors' judgement	Support for judgement
Random sequence generation (selection bias)	Low risk	Closed envelopes with the group assignment before surfery
Allocation concealment (selection bias)	Unclear risk	Envelopes used, but further concealment protection not mentioned
Blinding of participants and personnel (performance bias)	Unclear risk	Blinding not mentioned
Blinding of outcome assessment (detection bias)	Unclear risk	Blinding not mentioned
Incomplete outcome data (attrition bias)	Low risk	Two patients, one in each group, both with excellent functional results, didn't participate radiologic follow-up examination, data not analysed.
Selective reporting (reporting bias)	High risk	Addional outcome measure used but not described in the method section
Other bias	Unclear risk	There was insufficient information to judge the risk from other sources of bias.

Footnotes
