# Supplementary material for: Surgical management of chronic lateral ankle instability: a meta-analysis
Source: J Orthop Surg Res. 2018 Jun 25;13:159. doi: 10.1186/s13018-018-0870-6 (PMC6019311; doi:10.1186/s13018-018-0870-6)
Supplement: Supplementary file 6 — Characteristics of included studies 5 (PDF 91 kb) [file 13018_2018_870_MOESM6_ESM.pdf]

Characteristics of studies

Characteristics of included studies

Larsen 1990

**Methods**	Location: University of Copenhagen<br>Design: Prospective randomised trial<br>Method of randomisation: Enclosed slip designated the treatment by use of Geigy's random numbers<br>Assessor blinding: Radiographic evaluation at follow-up was blinded<br>Study period: 1980 to 1985<br>Follow-up: Mean 25 months, range 18 to 38 months<br>Intention-to-treat: No, 17 individuals in the dynamic repair group were excluded and not analysed, leaving 26 individuals for comparison.
**Participants**	99 patients with108 ankles were treated, only 82 patients (89 ankles) were included for comparison<br>46 man and 36 women, age range 17 to 49 years<br>Inclusion criteria:<br>(1) Recurring giving way of the ankle withour improvement after conservative treatment<br>(2) Manual and radiographic mechanical ankle instability<br>Exclusion criteria:<br>(1) Peroneus brevis tendon was too thin for splitting in operation<br>(2) Patients with open epiphyses<br>Loss to follow-up: 82 patients included for analysis, none were lost to follow-up
**Interventions**	(1) Dynamic tenodesis: the distal peroneus brevis tendon is split and the anterior part is used for a dynamic repairment<br>(2) Static tenodesis: the whole thickness of distal peroneus brevis tendon is used to make an static repairment of lateral ankle ligaments<br>Both groups underwent the same postoperative rehabilitation programme<br>Assigned: 99 participants (108 ankles): 43 participants (48 ankles) / 56 participants (60 ankles)<br><br>Analysed: 82 participants (89 ankles): 26 participants (29 ankles) / 56 participants (60 ankles)
**Outcomes**	(1) Evaluation scheme of the results: A 12 point score with 3 items: pain, degree of instability and decrease in strength was used for clinical assessment<br>(2) Functional balance: Ability to stand on one forefoot for ten seconds<br>(3) Mechanical stability by roentgenograms<br>(4) Postoperative complications: Nerve damage, DVT, ankle swelling, subsequent sprains, revision<br>(5) Postoperative sports activity
**Notes**	

Risk of bias table

Bias	Authors' judgement	Support for judgement
Random sequence generation (selection bias)	Low risk	Enclosed slip designated the treatment by use of Geigy's random numbers
Allocation concealment (selection bias)	Unclear risk	Envelopes used, but further concealment protection not mentioned
Blinding of participants and personnel (performance bias)	Unclear risk	Blinding of participants not mentioned

Blinding of outcome assessment (detection bias)	Low risk	Radiographic evaluation at follow-up was blinded
Incomplete outcome data (attrition bias)	High risk	Patients after randomisation excluded and not analysed
Selective reporting (reporting bias)	High risk	A second publication with other outcome measures of the same study population has been published
Other bias	Unclear risk	There was insufficient information to judge the risk from other sources of bias.

Footnotes
