# Supplementary material for: Surgical management of chronic lateral ankle instability: a meta-analysis
Source: J Orthop Surg Res. 2018 Jun 25;13:159. doi: 10.1186/s13018-018-0870-6 (PMC6019311; doi:10.1186/s13018-018-0870-6)
Supplement: Supplementary file 7 — Characteristics of included studies 6 (PDF 91 kb) [file 13018_2018_870_MOESM7_ESM.pdf]

Characteristics of studies

Characteristics of included studies

Porter 2015

**Methods**	Location: The Canberra Hospital, Canberra<br>Design: Randomized controlled trial<br>Method of randomisation: Randomization was performed using a single toss of a coin<br>Assessor blinding: No blinding<br>Study period: April 2009 to May 2010<br>Follow-up: 2 years<br>Intention-to-treat: There were no outliers in either group
**Participants**	There were 21 patients in the LARS group, 11 male and 10 female, mean age 26.1 years (range 16 – 43). There were 20 patients in the MBG group, 10 male and 10 female, mean age 24.0 years (range 16 – 41).<br>Inclusion criteria:<br>(1) Chronic instability (> 3 months) of ATFL and CFL<br>(2) Medically fit<br>(3) Physically active<br>(4) Failed non-operative treatment<br>(5) Skeletally mature<br>(6) Signed, informed consent<br>Exclusion criteria:<br>(1) Previous ankle surgery<br>(2) MBG contra-indicated<br>(3) Ankle fracture<br>(4) Diastasis<br>(5) MCL laxity<br>(6) >90 kg body mass<br>Loss to follow-up: No patients lost.
**Interventions**	MBG procedure: Anatomic repairment of the lateral ankle ligaments with three double-armed suture anchors<br>LARS procedure: Anatomic reconstruction of the lateral ankle ligaments with LARS AC 30 DB synthetic ligament<br>Both groups underwent the same post-operative rehabilitation programme.<br>Assigned: 20/21<br>Analysed: 20/21
**Outcomes**	(1) The foot and ankle outcome score (FAOS)<br>(2) Complications: Irritation of the peroneal tendons, wound complications, pseudoaneurysm
**Notes**	

Risk of bias table

**Bias**	**Authors' judgement**	**Support for judgement**
Random sequence generation (selection bias)	Low risk	Randomization was performed using a single toss of a coin
Allocation concealment (selection bias)	Unclear risk	Single toss of coin used, but further concealment protection not mentioned
Blinding of participants and personnel (performance bias)	High risk	Blinding was not possible, LARS procedure required two additional incision

Blinding of outcome assessment (detection bias)	High risk	Blinding was not possible, LARS procedure required two additional incision
Incomplete outcome data (attrition bias)	Low risk	There was no loss to follow-up
Selective reporting (reporting bias)	High risk	Addional outcome measure used but not described in the method section
Other bias	Unclear risk	There was insufficient information to judge the risk from other sources of bias

Footnotes
