# Supplementary material for: Surgical management of chronic lateral ankle instability: a meta-analysis
Source: J Orthop Surg Res. 2018 Jun 25;13:159. doi: 10.1186/s13018-018-0870-6 (PMC6019311; doi:10.1186/s13018-018-0870-6)
Supplement: Supplementary file 8 — Characteristics of included studies 7 (PDF 85 kb) [file 13018_2018_870_MOESM8_ESM.pdf]

Characteristics of studies

Characteristics of included studies

Rosenbaum 1999

**Methods**	Location: Westfalische Wilhelms-Universitat Munster<br>Design: Randomised trial<br>Method of randomisation: Not mentioned<br>Assessor blinding: Not mentioned<br>Study period: Not mentioned<br>Follow-up: 10±4 months<br>Loss to follow-up: Not mentioned, complete follow-up
**Participants**	10 participants in each group, all male, mean age 25 years<br>Inclusion criteria:<br>(1) Recurrent inversion injuries and pain in the ankle joint<br>(2) Radiological stress examination resulted in a talar tilt of more than 10° or in an anterior drawer sign of more than 10 mm<br>Exclusion criteria: Not mentioned<br>Loss to follow-up: Not mentioned
**Interventions**	(1) Evans group: Modified Evans tenodesis was used for reconstruction of the anterolateral ankle ligaments<br>(2) Periost group: Anatomic repairment of the anterolateral ankle ligaments with reinforcement with a periosteal flap<br>Both groups underwent the same post-operative rehabilitation programme<br>Assigned: 10/10<br>Analysed: 10/10
**Outcomes**	(1) Physical examination with manual evaluation of joint mobility<br>(2) Radiographic stress diagnostics in neutral ankle position with talar tilt, and anterior drawer measurements under a load application.
**Notes**	

Risk of bias table

**Bias**	**Authors' judgement**	**Support for judgement**
Random sequence generation (selection bias)	Unclear risk	The patients were assigned to one of two experimental groups after stratified randomization but no further details provided
Allocation concealment (selection bias)	Unclear risk	Allocation concealment not mentioned
Blinding of participants and personnel (performance bias)	High risk	Blinding not mentioned
Blinding of outcome assessment (detection bias)	High risk	Blinding not mentioned
Incomplete outcome data (attrition bias)	Low risk	There was no loss to follow-up
Selective reporting (reporting bias)	Low risk	Outcome measures the same in methods and results sections
Other bias	Unclear risk	There was insufficient information to judge the risk from other sources of bias

Footnotes
