# Supplementary material for: Surgical management of chronic lateral ankle instability: a meta-analysis
Source: J Orthop Surg Res. 2018 Jun 25;13:159. doi: 10.1186/s13018-018-0870-6 (PMC6019311; doi:10.1186/s13018-018-0870-6)
Supplement: Supplementary file 9 — Characteristics of excluded studies (PDF 90 kb) [file 13018_2018_870_MOESM9_ESM.pdf]

Characteristics of studies

Characteristics of excluded studies

Axelsen 1993

**Reason for exclusion**	No clinical outcome measures

Beynnon 2005

**Reason for exclusion**	Diagnosis study

Burks 1991

**Reason for exclusion**	Prevention study

Cesarone 2008

**Reason for exclusion**	About patients with vascular diseases

Chan 2009

**Reason for exclusion**	Not about chronic ankle instability

De Vries 2006

**Reason for exclusion**	Review

Derksen 2005

**Reason for exclusion**	Diagnosis study

Derksen 2015

**Reason for exclusion**	Diagnosis study

Docherty 2004

**Reason for exclusion**	Electromyography and kinesiology

Doeringer 2010

**Reason for exclusion**	Non-operative treatment

Faber 2004

**Reason for exclusion**	Not about chronic ankle instability

Giza 2013

**Reason for exclusion**	Cadaver study

Hayman 2010

**Reason for exclusion**	Prevention study

Hupperets 2010

**Reason for exclusion**	Cost-Benefit analysis

Janssen 2014

**Reason for exclusion**	Cost-Benefit analysis

Johannes 1993

**Reason for exclusion**	Non-operative treatment

Johnson 2007

**Reason for exclusion**	Diagnosis study

Konradsen 1999

**Reason for exclusion**	Review

Munk 1995

**Reason for exclusion**	Patients not randomisize

Okcu 2006

**Reason for exclusion**	Non-operative study

Pijnenburg 2003

**Reason for exclusion**	Operative versus conservative treatment

Povacz 1998

**Reason for exclusion**	Operative versus conservative treatment

Schuh 2016

**Reason for exclusion**	Cadaver study

Van Reijen 2014

**Reason for exclusion**	Prevention of ankle injuries

Zwipp 1991

**Reason for exclusion**	Operative versus conservative treatment

Zwipp 1992

**Reason for exclusion**	Operative versus conservative treatment

Footnotes
